# Supplementary material for: Projecting the end of the Zika virus epidemic in Latin America: a modelling analysis
Source: BMC Med. 2018 Oct 3;16:180. doi: 10.1186/s12916-018-1158-8 (PMC6169075; doi:10.1186/s12916-018-1158-8)
Supplement: Supplementary file 1 — Supporting Information for Projecting the end of the Zika virus epidemic in Latin America: a modelling analysis. (PDF 3772 kb) [file 12916_2018_1158_MOESM1_ESM.pdf]

Supporting Information

Projecting the end of the Zika virus epidemic in Latin America: a modelling analysis

Kathleen M O'Reilly, Rachel Lowe, W John Edmunds, Philippe Mayaud, Adam Kucharski, Rosalind M Eggo, Sebastian Funk, Deepit Bhatia, Kamran Khan, Moritz U Kraemar, Annelies Wilder-Smith, Laura C Rodrigues, Patricia Brasil, Eduardo Massad, Thomas Jaenisch, Simon Cauchemez, Oliver J Brady, Laith Yakob

S1. Compilation of data used in the analysis and selection of cities

Compilation of data

Member states of Latin America and the Caribbean (LAC) have been reporting the incidence of Zika cases to the Pan America Health Organization (PAHO). The data are released as *pdf* files, along with additional information such as ZIKV in pregnant women and other potential complications. As part of the analysis of the genomics of ZIKV across LAC [1] the Anderson Lab have carefully compiled cases of Zika from the PAHO website [2]. The data are available from January 2015 to August 2017. The process was repeated for PAHO files released up to August 2017. The US Centers for Disease Control also release detailed data for several countries [3]. We first checked the consistency of the incidence curves between PAHO-reported and CDC-reported incidence along with the frequency at which the data were updated on the CDC website. Based upon this, and the need for state-level analysis, only CDC data reported from Mexico were included in the analysis. The weekly number of cases of suspected and confirmed Zika cases for each state in Brazil was obtained from the Brazilian national infectious disease surveillance system (Sistema de Informacao de Agravos de Notificacao - SINAN). Details of this database, case diagnosis methods and changes in definitions have been previously described elsewhere [4].

Selection of cities in the analysis

Cities data were extracted from a publicly available database on world cities, compiled from the UN World Urbanisation Prospects [5, 6]. All cities greater than 750,000 were selected for analysis, as we felt that this balanced the capture of urban locations that may experience Zika and computational time that would be required to estimate parameters from a large mata-population dynamic transmission model. Not all countries within LAC have cities greater than 750,000, and for these countries (typically within the Caribbean) the largest city was selected fro analysis. This was repeated for Brazilian and Mexican states. The cities included in the analysis are provided in Table S1, grouped by country. The latitude and longitude of each city was used to infer seasonality curves of Zika transmission, by referencing the global map developed by Brady *et al.* [7] and also used by Bogoch *et al.* [8].

S2. Details of the mathematical model used in the analysis

As described in the main text we assumed that humans were classified by their infection status; susceptible, pre-infectious, infectious or recovered from ZIKV infection, corresponding to S, E, I R compartments in a mathematical model of infection. The connectivity between cities is a key component of epidemic spread, and is modelled assuming a meta-population, akin to the *commuter* model described in Keeling and Rohani [9]. Commuters live in one subpopulation (denoted by the ii subscript) but travel occasionally to another subpopulation (denoted by the ij subscript). From a standard SEIR model we consider the number of individuals of each type in each spatial class and the assumed movement between them:

$$\frac{dS_{ii}}{dt} = \mu - \beta_i(t)S_{ii}\frac{\sum_j I_{ij}}{\sum_j N_{ij}} - \sum_j l_{ji}S_{ii} + \sum_j r_{ji}S_{ji} - \mu S_{ii}$$
$$\frac{dS_{ij}}{dt} = -\beta_i(t)S_{ii}\frac{\sum_j I_{ij}}{\sum_j N_{ij}} + l_{ij}S_{ij} - r_{ij}S_{ij} - \mu S_{ij}$$
$$\frac{dE_{ii}}{dt} = \beta_i(t)S_{ii}\frac{\sum_j I_{ij}}{\sum_j N_{ij}} - \alpha E_{ii} - \sum_j l_{ji}E_{ii} + \sum_j r_{ji}E_{ji} - \mu E_{ii}$$
$$\frac{dE_{ij}}{dt} = \beta_i(t)S_{ii}\frac{\sum_j I_{ij}}{\sum_j N_{ij}} - \alpha E_{ij} + l_{ji}E_{ij} - r_{ji}E_{ij} - \mu E_{ij}$$
$$\frac{dI_{ii}}{dt} = \alpha E_{ii} - \gamma I_{ii} + \sum_j r_{ji}I_{ji} - \mu I_{ii}$$
$$\frac{dI_{ij}}{dt} = \alpha E_{ij} - \gamma I_{ij} - r_{ij}I_{ij} - \mu I_{ij}$$
$$\frac{dR_{ii}}{dt} = \gamma I_{ii} - \sum_j l_{ji}R_{ii} + \sum_j r_{ji}R_{ji} - \mu R_{ii}$$
$$\frac{dR_{ij}}{dt} = \gamma I_{ij} + l_{ij}R_{jj} - r_{ij}R_{ij} - \mu R_{ij}$$
$$\frac{dN_{ii}}{dt} = \mu - \sum_j l_{ji}N_{ii} + \sum_j r_{ji}N_{ji} - \mu N_{ii}$$
$$\frac{dN_{ij}}{dt} = -l_{ij}N_{jj} + r_{ij}N_{ij} - \mu N_{ij}$$

The parameters in the above set of equatons are summarized in Table 2, along with the values used in the analysis. To improve computational speed we only model the movement of pre-infectious individuals, as the movement of susceptible individuals does not impact the dynamics but consist of the bulk of movements. Infectious individuals were assumed to not move between cities, due to symptoms, although it is acknowledged that many infectious individuals are asymptomatic. The model was implemented in MatLab (R2017) and the output was analysed in R (version 3.3.3). Model code and outputs are available at https://github.com/kath-o-reilly/Zika-LAC-Outbreaks.

Movement models fitted to the Zika outbreak

The fit of five movement models were tested against the data on ZIKV outbreaks; i) a gravity-model with no exponential terms; ii) a gravity model with estimated exponential terms; iii) a radiation model; iv) a data-driven approach based on flight data; and v) a model of local radiation and flight

movements. Each model has different assumptions of the connectivity between each city, and will influence the rate of spread of ZIKV between them. There is currently limited information about the likely modes of human movement that influence the spread of ZIKV, but international movements via flights have been cited as a likely mode. For each model we assume that the probability of a pre-infectious individual moving from city i to city j follows the patterns specified in the following equations below. All probabilities are normalised such that $\sum_i \sum_j m_{ij} = 1$.

Model 1: gravity-model with no exponential terms

Movement is driven by city population sizes (n_i and n_j respectively) and the Euclidean distance between them (d_{ij}). The terms μ, ν and γ are exponential terms that affect the strength of size and distance on movement;

$$P(m_{ij}(g_1)) = \frac{n_i^{g^\mu} n_j^{g^\nu}}{d_{ij}^{g^\gamma}}$$

Model 2: gravity model with estimated exponential terms

The exponential terms (g^μ, g^ν and g^γ) of the gravity probability matrix $P(m_{ij}(g_2))$ were estimated from the data alongside the other parameters.

Model 3: radiation model

The radiation model was developed to account for local population movements being influenced by the need to travel until required resources (eg. employment) are met. The parameter s_{ij} described the population size within the circle defined by the locations of cities i and j (with coordinates $\{x_i, y_i\}$ and $\{x_j, y_j\}$ respectively);

$$P(m_{ij}(r)) = \frac{n_i n_j}{(n_i + s_{ij})(n_i + n_j + s_{ij})}$$

Population sizes were extracted from WorldPop, and to save on computational time if the d_{ij} >6000km $P(m_{ij}(r))$ was assigned a value of 10^{-5}.

Model 4: flight data Data from selected airports that correspond to the selected cities were extracted from the Global database of the International Air Transport Association (www.iata.org). Some cities (eg. Sao Paulo) have multiple airports and data from all airports associated with the city were extracted. For smaller (eg. Caribbean) countries all airports within the country were used in the analysis. The data of the total number of registered tickets between cities from January 2015 to December 2016 were aggregated to provide an estimate of the probability of moving from city i to city j, $P(m_{ij}(f))$. Movements in 2017 were assumed to follow the same patterns.

Model 5: radiation and flight data

The radiation ($P(m_{ij}(r))$) and flight ($P(m_{ij}(f))$) matrices were combined together assuming a mixture model with parameter η that describes the contribution of the radiation matrix to movement;

$$P(m_{ij}(a)) = \eta P(m_{ij}(r)) + (1 - \eta) P(m_{ij}(f))$$

Comparison of the different movement models

Figure S2 shows the effect of the three main (gravity, radiation and flight) movement models on movement out of Cali (Colombia) and Sao Paulo (Brazil). Both the gravity and radiation models predict that movement mostly occurs between cities within a short distance, and especially among large local cities for the radiation model. The flight data illustrates a different pattern, consisting of a higher probability of movements to large distant cities associated with tourism or important commerce

hubs.

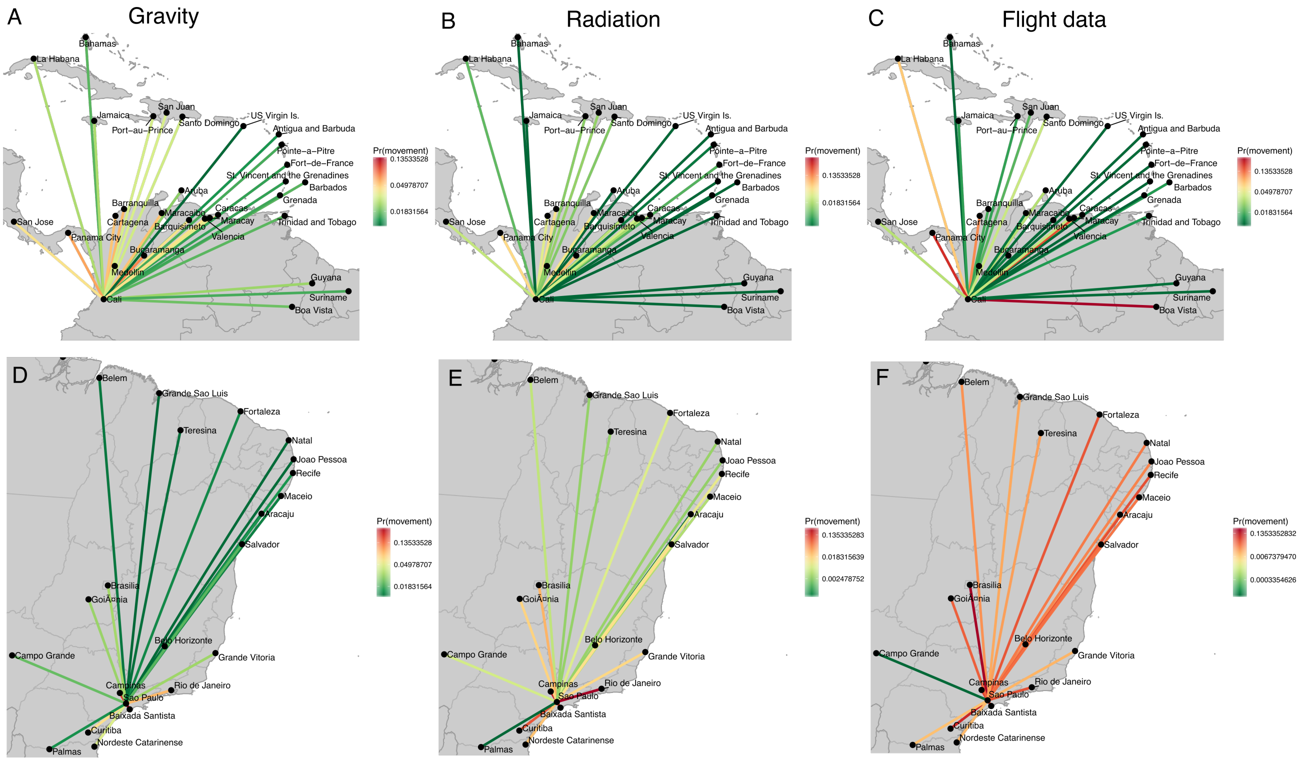

S2 Figure. Illustration of the three different movement models tested in the analysis; (A, D) gravity model, (B, E) radiation model and (C, F) flight data. The top row shows the estimated probability of movement out from Cali (Colombia) and the lower row shows the estimated probability of movement out from Sao Paulo (Brazil).

Additional details of using ABC-SMC to fit the data to the models

Let $m\{1, ..., M\}$ be a model parameter that specifies the movement model, where M is the total number of models. The model-specific parameters are denoted by $\theta(m) = (\theta(m)^{(1)}, ..., \theta(m)^{(k_m)}), m = 1, ..., M$, where k_m denotes the number of parameters in model m. Following the algorithm given in Toni et al [16], ABC was used to approximate the posterior distribution $\pi(\theta|x)$ based on the distance between the model outputs and the data being below a specified tolerance value $d(x_0, x^*) \leq \epsilon$. The distance function is a comparison of the timing of the peak of ZIKV epidemics within each of the geographical units in the analysis (countries of Latin America and the Caribbean, and States within Brazil and Mexico) and the modelled cities, ie. $X_{p,i}$ describes the reported peak for each geographical unit in the data and $Z_{p,i}$ describes the estimated peak from the mathematical modelling (where city timeseries Z_j were summed to provide Z_i). To account for multiple cities being present within some geographical units, the model and data were weighted by the number of cities (n_i) within each geographical unit $d(x_0, x^*) = \sum(X_{p,i} - Z_{p,i})^2/n_i^2$.

We start by running a set of particles ($n = 100$) where each particle simulates the ZIKV epidemic assuming model m. The parameters of each model are initially selected from the priors specified in Table 2, m^* from $\pi(m)$ and θ^{**} from $\pi(\theta(m^*))$. 1000 iterations of each particle were run, where parameter sets were randomly picked from the range in Table 2 and the accpeted parameter sets were used to inform the multvariate normal distribution used in the second round. For the second round, 100 particles were again selected, but particles were sampled using multinomial distribution

with probabilities from the previous round. Parameter sets of a selected model (ie. θ^{**}) were sampled from a pertubation kernel based on the accepted parameter sets from the previous population and the estimated covariance matrix ($\theta^{**} = K_t(\theta|\theta^*)$). An ABC-MCMC algorithm was used to approximate the posterior distribution, ie. θ^{**} was accepted with probability $\alpha = min(1, \frac{\pi(\theta^{**})q(\theta_i|\theta^{**})}{\pi(\theta_i)|q(\theta^{**}|\theta_i)})$ when $d(x_0, x^*) \leq \epsilon$, otherwise $\theta_{i+1} = \theta_i$. 5,000 iterations of each model were run. The mean and 95% credible intervals (CrI) of each parameter were estimated from the particles where the preferred model was selected.

Bayes Factors were used for model selection. From the ABC-SMC we have an approximation of the marginal posterior distribution, based on the particles with accepted parameter sets. Using the particles from the second population Bayes Factors were estimated using $BF_{12} = \frac{P(m_1|x)/P(m_2|x)}{P(m_1)/P(m_2)}$. The best fitting model was based on interpretation of the Bayes Factors provided by Kass and Raftery [17].

S3. Vectorial capacity modelling for ZIKV transmisison

A species distribution modelling approach was used to establish an empirical relationship between the probability of ZIKV occurrence and environmental conditions. For ZIKV to be transmitted the vector *Aedes aegypti* needs to persist at a sufficient density and environmental conditions needed to favour virus replication within the mosquito [7]. Time-varying maps of *Aedes aegypti* relative probability of occurrence [8] were assumed to be proportional to mosquito abundance (m). These were then combined with temperature suitability models which detail vectorial capacity excluding mosquito abundance and human-mosquito effective contact rate for dengue ($\frac{p^n}{-ln(p)}$) [7]. We made the assumption that viral extrinsic incubation period of dengue virus was equal to that of ZIKV. This combination of abundance and temperature suitability models gave vectorial capacity excluding effective human-mosquito contact rate: $z_i(t)$. This estimate was extrapolated to vectorial capacity (VC) model as follows. The mathematical model of ZIKV (described in *S2*) encapsulates ZIKV transmission using the coefficient $\beta_i(t)$, and we assume that $\beta_i(t) = z_i(t) \times c$, where c is unknown and encapsulates vector competence and vector biting rate (effective human contact rate, $c = ba^2$). In the parameter estimation procedure we indirectly estimate c from the data by estimating the maximum value of $\overline{R_{0,i}}$ within all the modelled cities. $\overline{R_{0,i}}$ is the average value of the basic reproduction number for city i, ie. $\overline{R_{0,i}} = \sum R_{0,i}(t)/n$, where $R_{0,i}(t) = \frac{\beta_i(t)\alpha}{(\alpha+\mu)(\gamma+\mu)}$. In summary;

$$VC = ba^2.m.\frac{p^n}{-ln(p)}$$

Where:

b = vector competence (inferred from case data)

a = vector biting rate (inferred from case data)

m = number of mosquitoes per human (modelled using ecological niche models from Bogosch *et al.* [8])

p = daily probability of survival of the vector (modelled using temperature suitability models from Brady *et al.* [7])

n = number of days to complete extrinsic incubation period (modelled using temperature suitability models from Brady *et al.* [7])

S4. Testing for the impact of surveillance sensitivity on the estimated timing of the peak in incidence

A concern with the data quality of the Zika epidemic is that surveillance for ZIKV cases may have substantially increased from January 2016 (due to an increasing awareness about the epidemic) and may have reduced after December 2016 (due to reduced reporting of cases and less media coverage on the epidemic). Moreover, Zika-specific surveillance systems in LAC became operational in December 2015, and PAHO reported incidence only begins in January 2016 despite cases being reported in several countries prior to this time. To test the impact of changes in surveillance within each country we applied a scaling factor to each time series for observations prior to March 2017 and after December 2016. For each country time series we have the weekly number of reported Zika cases ($\mathbf{X} = \{X_t, t = 1, ..., T\}$) and we apply scaling factors to produce an updated time series ($\mathbf{Y} = \{Y\}_t, t = 1, ..., T\}$);

$$Y_t = \begin{cases} aX_t & \text{if } t < 2016.25 \\ X_t & \text{if } 2016.25 \leq t < 2017 \\ bX_t & \text{if } t \geq 2017 \end{cases}$$

We then identified the number of countries where the peak incidence would change as a result of the change in reporting. We varied the scaling factor within plausible value to determine the effects on estimated timing of the peak. Figure S1 shows that surveillance prior to March 2016 would need to be 25% or less than 2016 intensity for 10 or more observations to have a change in the estimated peak, making the peak incidence quite robust to changes in surveillance. The peak in incidence would change for 13 countries if surveillance had reduced in 2017, and increasing surveillance would affect the peak estimate for 2 countries, again illustrating that peak incidence is a useful summary statistic in this setting.

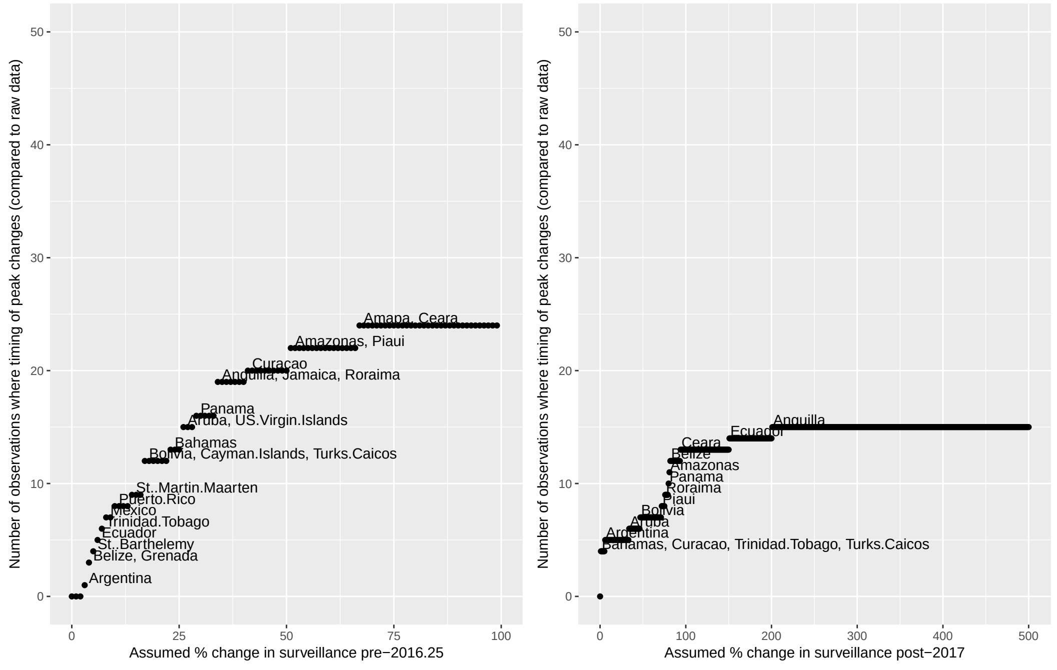

S4 Figure. Impact of assuming a step-change in surveillance (left) prior to March 2016 and (right) after December 2016, on the estimated peak in ZIKV cases.

S5. Results from the parameter estimation

Modelling of the first population of MCMC particles assume that each model had an equal probability of fitting the data. The posterior probability that model m would be selected was used as the prior for the second population, and the probability is shown in Table 1 of the main text. A selection (n=5) of particles and their MCMC chains from the second population is shown in Figure S3. To reduce auto-correlation the chains were thinned to every tenth iteration. We illustrate the maximal value of R_0 and the estimated leave rate from the migration matrix. Whilst the chains seem quite stationary, a correlation plot between R_0 and the leave rate show a strong negative correlation when R_0 reaches high values. Summary values of the parameters are provided in Table 3.

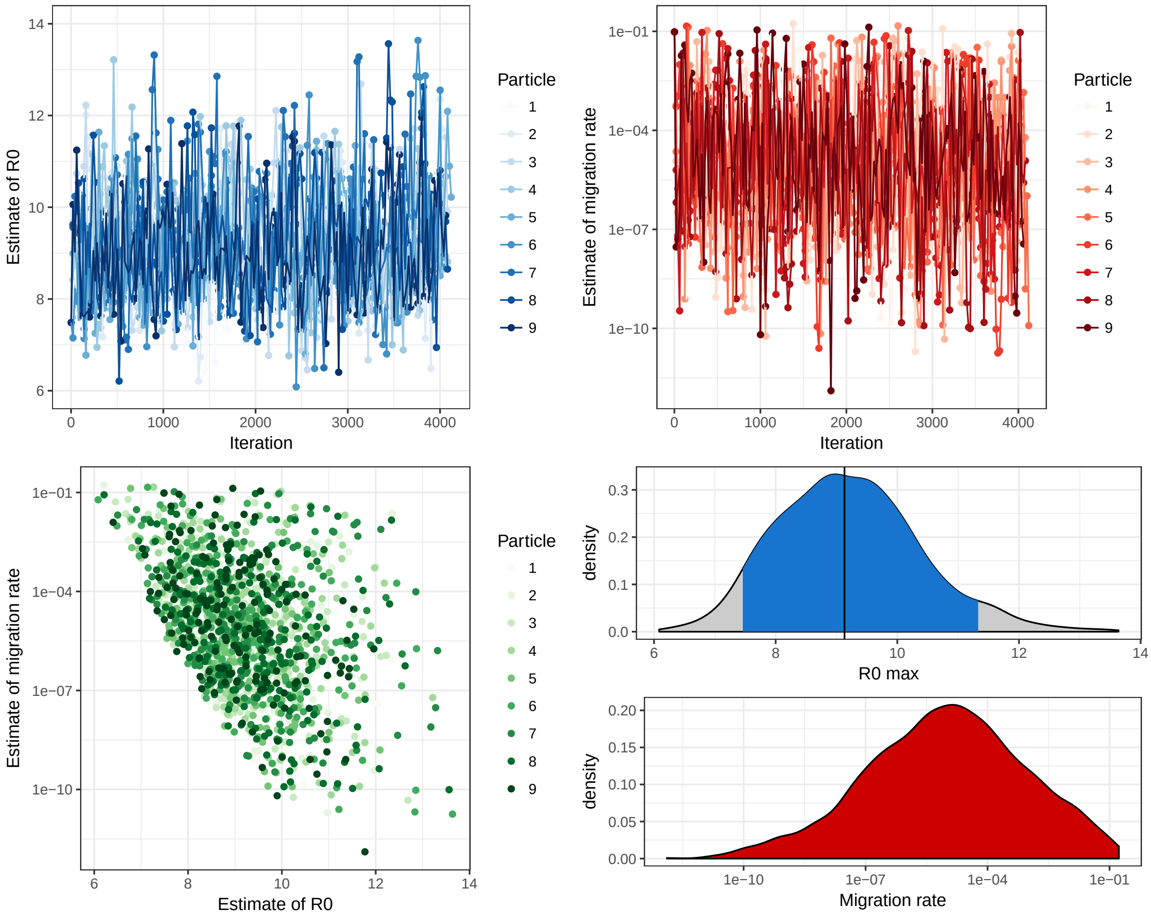

S5 Figure. MCMC output of the parameter estimation from 9 particles where the gravity model was selected.

S6. Analysis of data and model comparisons for Brazilian States

Data were available on suspected and confirmed Zika cases within Brazil at a State level, enabling a state-level analysis of the epidemic. All cities greater than 750,000 were included in the analysis. For states that do not have cities of this size, the largest city was also included in the model (Figure S6a). A more detailed comparison of the peak timing of ZIKV reported and estimated is provided in Figure S6b, illustrating that for most states the peak timing is predicted well by the model. The peak timing for Paraiba, Amazonas, Ceara, Piaui and Roraima were predicted to be earlier than reported, and in these States (with the exception of Paraiba) the peak was reported to be in 2017.

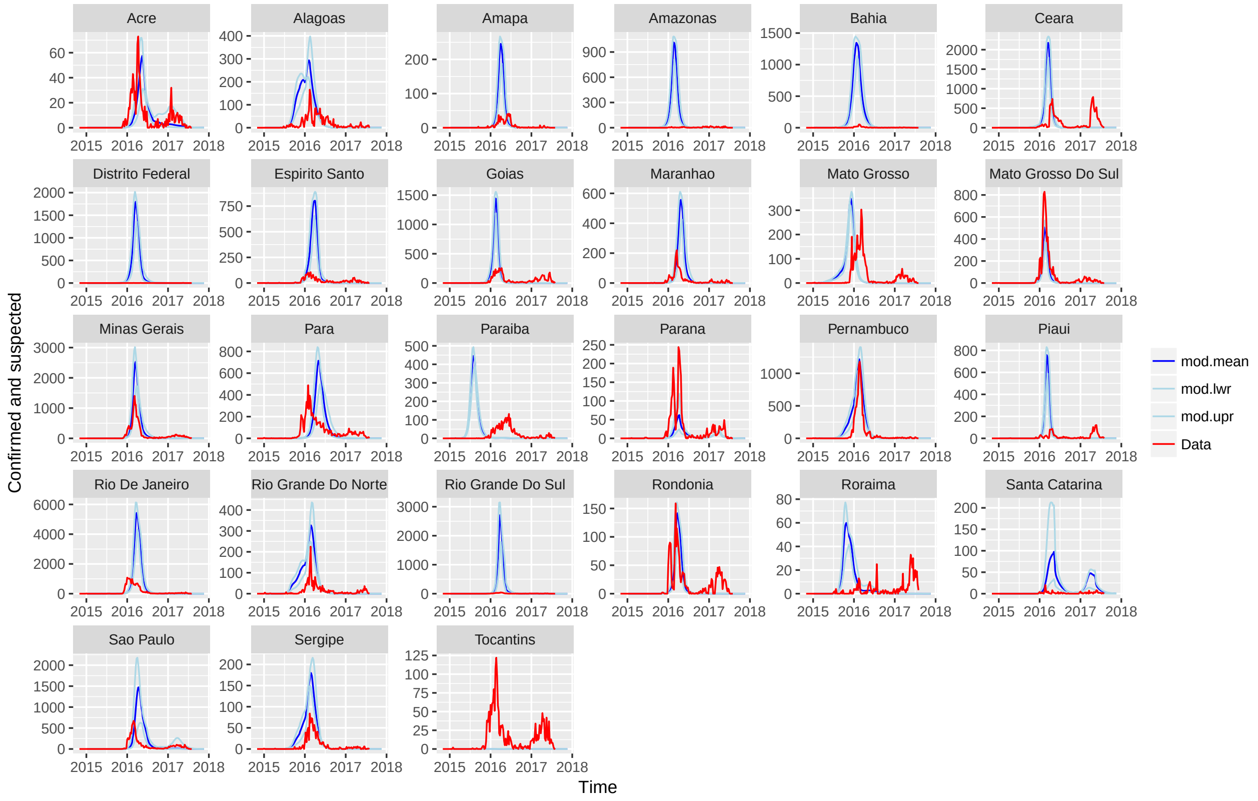

S6a Figure. Comparison of reported Zika cases to the median (and 95% CI) estimate of cases from the mathematical model for States within Brazil. Note that the y-axes for each subplot are set to different scales to improve the clarity of visualising the data.

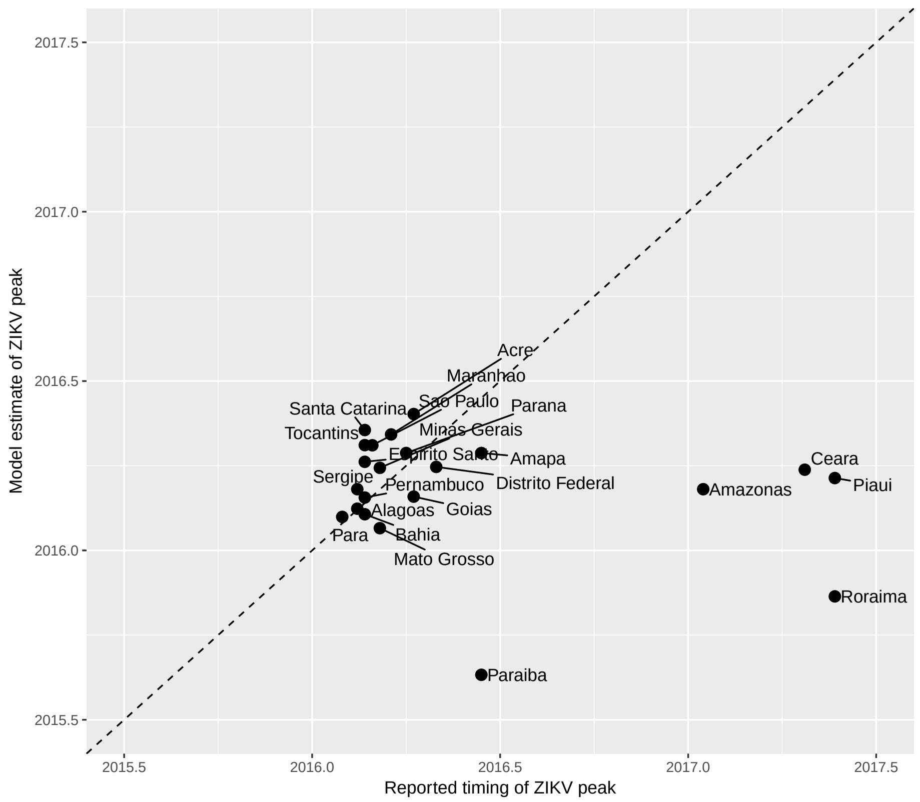

S6b Figure. Comparison of peak in Zika cases to the median estimate from the mathematical model, where the peak is compared between all States. The dashed line indicates perfect agreement.

S7. Analysis of data and model comparisons for Mexican States

Data were available on suspected and confirmed Zika cases within Mexico at a State level, enabling a state-level analysis of the epidemic. All cities greater than 750,000 were included in the analysis, and because cases reported outside of these states were low the analysis focussed on only these states (Figure S5).

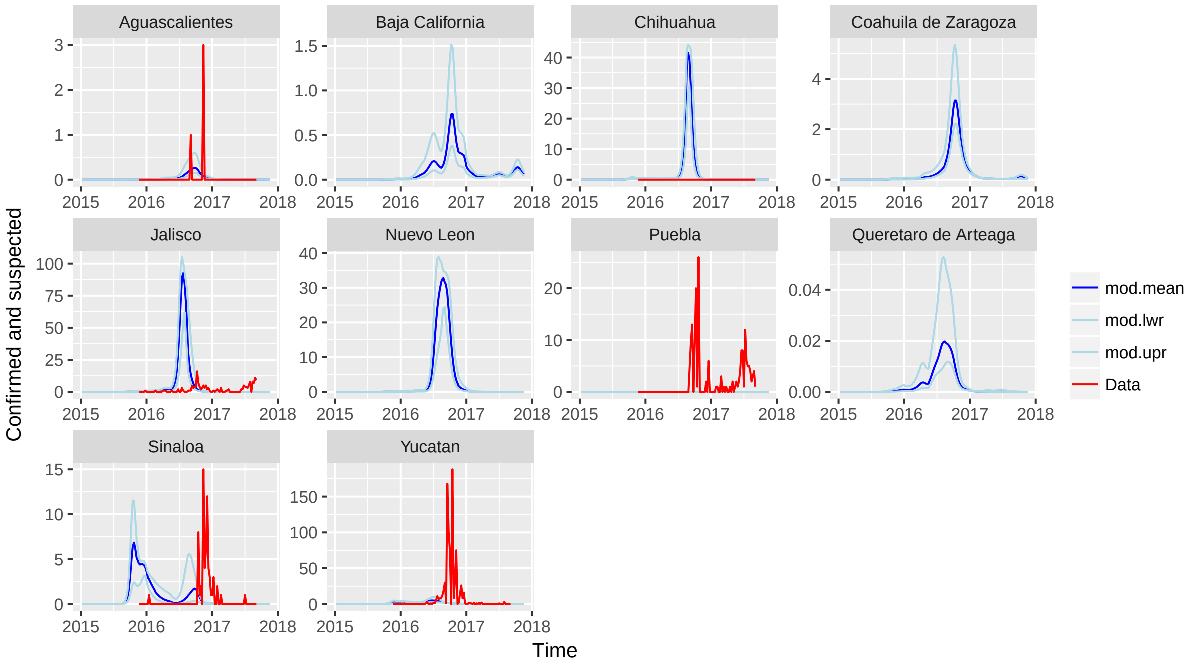

S7 Figure. Comparison of reported Zika cases to the median (and 95% CI) estimate of cases from the mathematical model for States within Mexico. Only states that reported ZIKV cases are plotted. Note that the y-axes for each subplot are set to different scales to improve the clarity of visualising the data.

S8. Testing the assumptions of population movements in the simulations

For the majority of the simulations, we make a simplifying assumption that individuals that are exposed are the only individuals that travel; movement of susceptibles were ignored as they will not impact on simulations (and are computationally expensive to include), and infectious individuals were also ignored as we assumed that ZIKV symptoms would limit travel. However, a majority of infectious individuals will not have symptoms and so this assumption may be invalid. To test the impact of this assumption we ran a small number of simulations assuming that infectious individuals also travelled and compared the parameter estimates and 2018 projections to those included in the main text. The 70 model runs were selected based on having equivalent fit to the data. Figure S8A illustrates that the parameter estimates of R_0 and the migration rate are not heavily impacted by whether infectious individuals move or not, although there is perhaps a smaller parameter space that results in an equivalent fit. Figure S8B illustrates that the projected incidence for 2018 for each city and Caribbean country included in the analysis were also not heavily impacted by this assumption. For a majority of cities the simulations under both assumptions projected a similar number of infections, but a small number (13 of 90 cities) of cities a higher number of infected individuals were projected in 2018 when simulations assume that infectious individuals move.

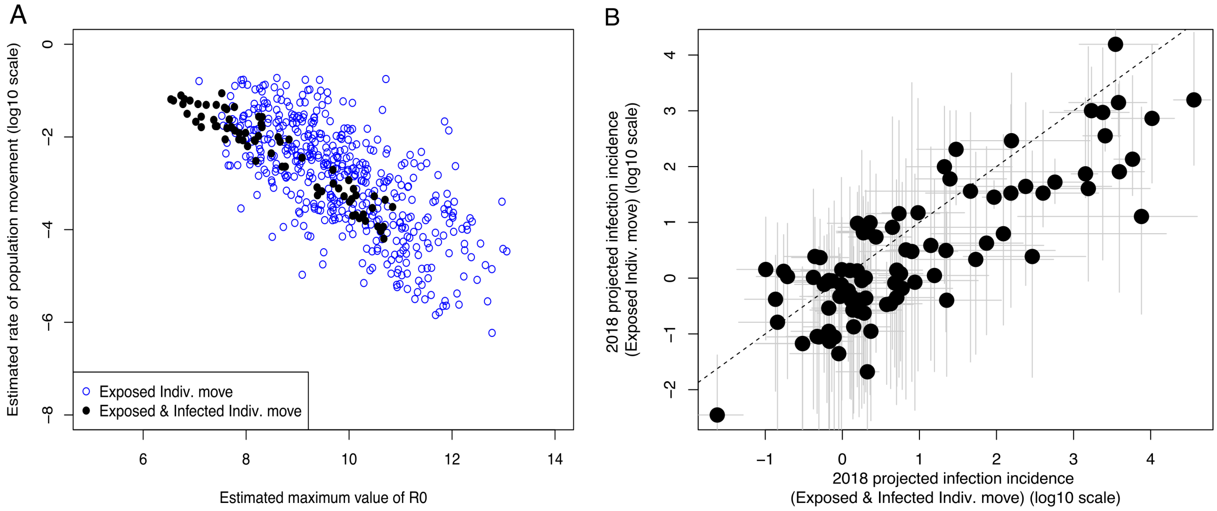

S8 Figure. Parameter estimates (A) and projected incidence (B) for the 90 cities and Caribbean countries included in the analysis under different assumptions about the movement of infectious individuals. The grey lines in B indicate 90% CI and the dashed line indicates perfect agreement.

References

[1] Grubaugh, N. D. *et al.* Genomic epidemiology reveals multiple introductions of Zika virus into the United States. *Nature* **546**, 401–405 (2017). URL http://www.nature.com/doifinder/10.1038/nature22400.

[2] Andersen, K. PAHO Zika numbers - Andersen Lab (2017). URL https://andersen-lab.com/paho-zika-cases/.

[3] Centers for Disease Control and Prevention (CDC). GitHub repository - cdcepi/zika (2017).

[4] de Oliveira, W. K. *et al.* Infection-related microcephaly after the 2015 and 2016 Zika virus outbreaks in Brazil: a surveillance-based analysis. *Lancet (London, England)* **390**, 861–870 (2017). URL http://linkinghub.elsevier.com/retrieve/pii/S0140673617313685http://www.ncbi.nlm.nih.gov/pubmed/28647172.

[5] UN. World Urbanization Prospects - Population Division - United Nations. URL https://esa.un.org/unpd/wup/.

[6] Nordpil. World database of large urban areas, 1950-2050. URL https://nordpil.com/resources/world-database-of-large-cities/.

[7] Brady, O. J. *et al.* Global temperature constraints on Aedes aegypti and Ae. albopictus persistence and competence for dengue virus transmission. *Parasites & Vectors* **7**, 338 (2014). URL http://parasitesandvectors.biomedcentral.com/articles/10.1186/1756-3305-7-338.

[8] Bogoch, I. I. *et al.* Potential for Zika virus introduction and transmission in resource-limited countries in Africa and the Asia-Pacific region: a modelling study. *The Lancet Infectious Diseases* **16**, 1237–1245 (2016). URL http://dx.doi.org/10.1016/S1473-3099(16)30270-5.

[9] Keeling, M. J. & Rohani, P. *Modeling Infectious Diseases in Humans and Animals* (Princeton University Press, 2008).

[10] Kucharski, A. J. *et al.* Transmission Dynamics of Zika Virus in Island Populations: A Modelling Analysis of the 2013 and 2014 French Polynesia Outbreak. *PLoS Neglected Tropical Diseases* **10**, 1–15 (2016). URL http://dx.doi.org/10.1371/journal.pntd.0004726.

[11] Campos, G. S., Bandeira, A. C. & Sardi, S. I. Zika Virus Outbreak, Bahia, Brazil. *Emerging Infectious Diseases* **21**, 1885–1886 (2015). URL http://www.ncbi.nlm.nih.gov/pubmed/26401719http://www.pubmedcentral.nih.gov/articlerender.fcgi?artid=PMC4593454http://wwwnc.cdc.gov/eid/article/21/10/15-0847{_}article.htm.

[12] Lessler, J. *et al.* Times to key events in the course of zika infection and their implications for surveillance: A systematic review and pooled analysis. *bioRxiv* (2016). URL https://www.biorxiv.org/content/early/2016/03/02/041913. https://www.biorxiv.org/content/early/2016/03/02/041913.full.pdf.

[13] Venturi, G. *et al.* An autochthonous case of Zika due to possible sexual transmission, Florence, Italy, 2014. *Eurosurveillance* **21**, 30148 (2016). URL http://www.ncbi.nlm.nih.gov/pubmed/26939607http://www.eurosurveillance.org/ViewArticle.aspx?ArticleId=21395.

[14] Bank, W. Life expectancy at birth (2017). URL https://data.worldbank.org/indicator/SP.DYN.LE00.IN.

[15] Gog, J. R. *et al.* Spatial Transmission of 2009 Pandemic Influenza in the US. *PLoS Computational Biology* **10**, e1003635 (2014). URL http://www.ncbi.nlm.nih.gov/pubmed/24921923http://www.pubmedcentral.nih.gov/articlerender.fcgi?artid=PMC4055284http://dx.plos.org/10.1371/journal.pcbi.1003635.

[16] Toni, T., Welch, D., Strelkowa, N., Ipsen, A. & Stumpf, M. P. H. Approximate Bayesian computation scheme for parameter inference and model selection in dynamical systems. *Journal of the Royal Society, Interface* **6**, 187–202 (2009). URL http://www.ncbi.nlm.nih.gov/pubmed/19205079http://www.pubmedcentral.nih.gov/articlerender.fcgi?artid=PMC2658655.

[17] Kass, R. E. & Raftery, A. E. Bayes Factor (1995).

Table 1: Country and State (in Brazil and Mexico) of cities included in the analysis

Country	State	Cities included in analysis
Antigua and Barbuda		
Argentina		Buenos Aires, Cordoba, Mendoza, Rosario, San Miguel de Tucuman
Aruba		
Bahamas		
Barbados		
Belize		
Bolivia		Santa Cruz
Brazil	Acre	Rio Branco
Brazil	Alagoas	Maceio
Brazil	Amapa	Macapa
Brazil	Amazonas	Manaus
Brazil	Bahia	Salvador
Brazil	Ceara	Fortaleza
Brazil	Distrito Federal	Brasilia
Brazil	Espirito Santo	Grande Vitoria
Brazil	Goias	Goiania
Brazil	Maranhao	Grande Sao Luis
Brazil	Mato Grosso	Cuiaba
Brazil	Mato Grosso do Sul	Campo Grande
Brazil	Minas Gerais	Belo Horizonte
Brazil	Para	Belem
Brazil	Paraiba	Joao Pessoa
Brazil	Parana	Curitiba
Brazil	Pernambuco	Recife
Brazil	Piaui	Teresina
Brazil	Rio de Janeiro	Rio de Janeiro
Brazil	Rio Grande do Norte	Natal
Brazil	Rio Grande do Sul	Porto Alegre
Brazil	Rondonia	Porto Velho
Brazil	Roraima	Boa Vista
Brazil	Santa Catarina	Florianopolis, Nordeste Catarinense
Brazil	Sao Paulo	Baixada Santista, Campinas, Sao Paulo
Brazil	Sergipe	Aracaju
Brazil	Tocantins	Palmas
Colombia		Barranquilla, Bucaramanga, Cali, Cartagena, Medellin
Costa Rica		San Jose
Cuba		La Habana
Curacao		Willemstad
Dominican Republic		Santo Domingo
Ecuador		Guayaquil
El Salvador		San Salvador
French Guiana		Cayenne
Grenada		Grenada
Guadeloupe		Pointe-a-Pitre
Guatemala		Guatemala City
Guyana		Guyana
Haiti		Port-au-Prince
Honduras		Tegucigalpa
Jamaica		Jamaica
Martinique		Fort-de-France
Mexico	Aguascalientes	Aguascalientes
Mexico	Baja California	Mexicali, Tijuana
Mexico	Chihuahua	Chihuahua, Ciudad Juarez
Mexico	Coahuila de Zaragoza	Saltillo, Torreon
Mexico	Jalisco	Guadalajara
Mexico	Nuevo Leon	Leon de los Aldamas, Monterrey
Mexico	Puebla	Puebla
Mexico	Queretaro de Arteaga	Querataro
Mexico	Sinaloa	Culiacan
Mexico	Yucatan	Merida
Nicaragua		Managua
Panama		Panama City
Paraguay		Asuncion
Peru		Lima
Puerto Rico		San Juan
Saint Vincent and the Grenadines		
Suriname		
Trinidad and Tobago		
United States Virgin Islands		Charlotteville
Venezuela		Barquisimeto, Caracas, Maracaibo, Maracay, Valencia

Parameter	Definition	Values used in analysis	Source
	Main parameters		
$\beta_i(t)$	Time-varying transmission rate	Varied across settings	Estimated from data
$1/\alpha$	Intrinsic incubation period	5 days	[10] [11] [12] [13]
$1/\gamma$	Human infectious period	20 days	[10] [11] [12] [13]
$1/\mu$	Human life expectancy	75 years	[14]
l_{ij}	Rate that humans leave city j and commute to city i	$P(m_{ij})\kappa_m$	Estimated from data
r_{ij}	Rate of return from i to j	0.001 per day	[9]
	Model-specific parameters	*Range of values tested in ABC-SMC algorithm*	
κ_g, κ_r and κ_f	Scaling parameters for *gravity*, *radiation* and *flight* model	All varied between 10^{-10} and 10^{-1}	
g^μ, g^ν and g^γ	Exponents of the gravity model	Varied from $[1,5]$, $[0.01,1]$ and $[0.1,1]$ respectively	[15]

Table 2: Parameters used in the mathematical model for Zika virus transmission.

Parameters estimated in the model	Median value (95% CrI)
$R_{0,max}$	7.00 (5.61, 8.51)
leave rate	0.03 (0.0003, 0.19)
g^μ	2.1 (2.0, 2.2)
g^ν	0.36 (0.1, 0.48)
g^γ	0.12 (0.5, 0.90)

Table 3: Parameter estimates (and 95% credible intervals) from the gravity model fitted to the ZIKV data and mathematical model
